# Efficient Production of Poly(Cyclohexene Carbonate) via ROCOP of Cyclohexene Oxide and CO_2_ Mediated by NNO-Scorpionate Zinc Complexes

**DOI:** 10.3390/polym12092148

**Published:** 2020-09-21

**Authors:** Sonia Sobrino, Marta Navarro, Juan Fernández-Baeza, Luis F. Sánchez-Barba, Agustín Lara-Sánchez, Andrés Garcés, José A. Castro-Osma, Ana M. Rodríguez

**Affiliations:** 1 Centro de Innovación en Química Avanzada (ORFEO-CINQA), Departamento de Química Inorgánica, Orgánica y Bioquímica, Universidad de Castilla-La Mancha, Campus Universitario, 13071 Ciudad Real, Spain; Sonia.Sobrino@uclm.es (S.S.); Juan.FBaeza@uclm.es (J.F.-B.); Agustin.Lara@uclm.es (A.L.-S.); JoseAntonio.Castro@uclm.es (J.A.C.-O.); AnaMaria.RFdez@uclm.es (A.M.R.); 2 Departamento de Biología y Geología, Física y Química Inorgánica, Universidad Rey Juan Carlos, Móstoles, 28933 Madrid, Spain; marta.navarro.sanz@urjc.es (M.N.); andres.garces@urjc.es (A.G.)

**Keywords:** scorpionate zinc complexes, ring-opening copolymerization (ROCOP), CO_2_ fixation, poly(cyclohexene carbonate) production

## Abstract

New mono- and dinuclear chiral alkoxide/thioalkoxide NNO-scorpinate zinc complexes were easily synthesized in very high yields, and characterized by spectroscopic methods. X-ray diffraction analysis unambiguously confirmed the different nuclearity of the new complexes as well as the variety of coordination modes of the scorpionate ligands. Scorpionate zinc complexes **2**, **4** and **6** were assessed as catalysts for polycarbonate production from epoxide and carbon dioxide with no need for a co-catalyst or activator under mild conditions. Interestingly, at 70 °C, 10 bar of CO_2_ pressure and 1 mol % of loading, the dinuclear thioaryloxide [Zn(bpzaepe)_2_{Zn(SAr)_2_}] (**4**) behaves as an efficient and selective one-component initiator for the synthesis of poly(cyclohexene carbonate) via ring-opening copolymerization of cyclohexene oxide (CHO) and CO_2_, affording polycarbonate materials with narrow dispersity values.

## 1. Introduction

Over the last decade the conversion of carbon dioxide (CO_2_) into commercially viable commodities has attracted great interest in the scientific community, since carbon dioxide represents a real alternative carbon feedstock for a sustainable chemical industry [1,2].

**Scheme 1:**
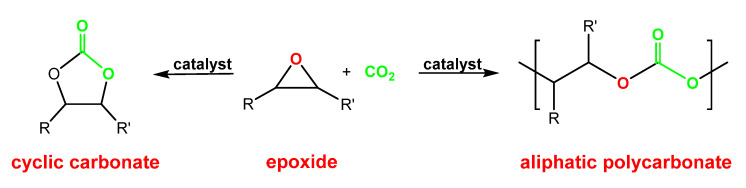
Synthesis of cyclic and polycarbonates.

CO_2_ is an attractive C-1 renewable building block [3] given its abundance in nature, low cost, non-toxicity, lack of colour and redox activity. Many chemical transformations are possible for this unsaturated molecule; however, the selective production of cyclic carbonates or polycarbonates through the cycloaddition or the ring-opening copolymerization (ROCOP) of CO_2_ with epoxides, respectively, (see Scheme 1) is gaining high attention as a 100% atom-economy route to convert waste CO_2_ into valuable materials [4,5].

Thus, cyclic carbonates present important applications as electrolytes, engineering plastics, solvent, fuel additives, and precursors to fine chemicals [6], whereas polycarbonates incorporate very smart physical features, such as durability, moldability, lightness, transparency and impact resistance [7], in addition to their biodegradability and biocompatibility that make them highly attractive in the biomedical field [8,9].

The structures of the resulting polycarbonates, which can include up to 50% of carbon dioxide in the polymer backbone, will determine their future applications. For instance, non-isocyanate polyurethanes (NIPUs) can be prepared employing initially low molar mass hydroxyl-telechelic polycarbonates [10,11], whereas higher molar mass CO_2_-derived polycarbonates find numerous applications as engineering polymers, packaging plastics, elastomers, adhesives and coatings [12].

Nevertheless, given the high thermodynamic stability and kinetic inertness of the CO_2_ molecule [13], the environmental and economical viability of the ROCOP depends on the capability of the catalytic system to avoid high temperatures and pressures [14], which should be able, in turn, to operate at ambient temperatures and pressures [15] as well as controlling rates and polymer molecular weight and composition [16]. In this context, very active and selective metal-based catalysts have been successfully developed for the ROCOP of carbon dioxide and epoxides, frequently in the presence of a nucleophile as co-catalyst, with zinc [17,18,19,20,21,22,23,24,25], chromium [26,27], cobalt [28,29], iron [30,31], rare earth metals [32,33] and aluminum [34,35] as dominating metals in this field, although non-metal and organocatalyst systems have been also recently reported [36]. Similarly, very efficient bimetallic systems have been also reported for the selective copolymerization of epoxides and carbon dioxide, in which the epoxide is activated by one metal, while the attacking nucleophile is provided by the second centre [37,38].

**Chart 1:**
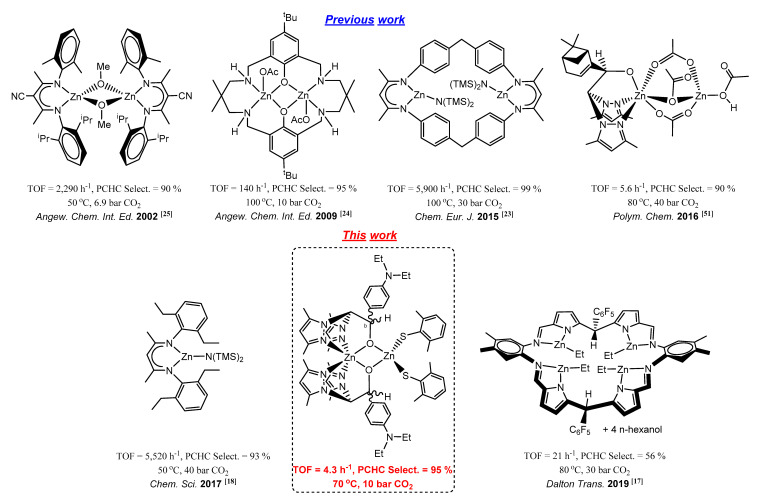
Representative zinc-based catalysts for the synthesis of poly(cyclohexene carbonate) via ROCOP of cyclohexene oxide and CO_2_.

Considering the current potential large-scale production of aliphatic polycarbonates by several companies [39,40,41], the employment of biocompatible metals such as zinc [42,43] is highly desirable to avoid potential health issues related to the toxicity of several metal-based residues in the isolated copolymers [44,45]. In particular, very active zinc-based catalysts in the absence of co-catalyst have been described [17,18,19,20,21,22,23,24,25] for polycarbonate production, some of them including alkoxide, amide, alkyl and acetate ligands as nucleophile in a coordination-insertion mechanism (see Chart 1).

On the other hand, a key point in controlling both reactivity and product selectivity of the catalyst is the nature of the ligand framework around the Lewis acidic metal centre. In this context, our research group has extensively studied the coordination chemistry of novel heteroscorpionate ligands [46], and a wide variety of applications have been reported in homogenous catalysis. For instance, we have designed versatile NNO-scorpionate alkyl and acetate zinc complexes that behaved as single-component initiators for the ring-opening co- and polymerization of cyclic esters [47,48,49,50], and for the ring-opening copolymerization of cyclohexene oxide with CO_2_ [51], respectively, as well as dinuclear NNO-scorpionate alkyl zinc analogues that displayed excellent performances in the cycloaddition of epoxide with carbon dioxide under mild and solvent-free conditions [52]. Now, we take on the challenge of designing more efficient cooperative homodinuclear NNO-scorpionate zinc catalysts containing thioalkoxide auxiliary ligands to enhance catalytic activity for CO_2_ fixation into the selective production of polycarbonates under milder conditions.

Hereby, we report the design of multinuclear scorpionate organo-zinc complexes and their use as efficient catalysts for the ring-opening copolymerization of cyclohexene oxide and carbon dioxide to produce poly(cyclohexene carbonate) under mild conditions.

## 2. Materials and Methods

### 2.1. Materials

All manipulations were carried out under a nitrogen atmosphere using standard Schlenk techniques or a glovebox. Solvents were predried over sodium wire and distilled under nitrogen from sodium (toluene and *n*-hexane) or sodium-benzophenone (THF and diethyl ether). Deuterated solvents were stored over activated 4 Å molecular sieves and degassed by several freeze-thaw cycles. The zinc alkyls ZnR_2_ (R = Me, Et) and 2,6-dimethylphenol or 2,6-dimethylthiophenol were used as purchased (Sigma-Aldrich, St. Louis, MO, USA). The starting materials bpzampeH [53], and bpzaepeH [53] were also prepared according to literature procedures.

### 2.2. Experimental

#### 2.2.1. Nuclear Magnetic Resonance Spectroscopy (NMR)

NMR spectra were recorded on Bruker Advance Neo 500 (Billerica, MA, USA) (^1^H NMR 500 MHz and ^13^C NMR 125 MHz) spectrometer and referenced to the residual deuterated solvent. The NOESY-1D spectra were recorded with the following acquisition parameters: irradiation time 2 s and number of scans 256, using standard VARIAN-FT software. Two-dimensional NMR spectra were acquired using standard VARIAN-FT software and processed using an IPC-Sun computer.

#### 2.2.2. Elemental Analysis

Microanalyses were performed with a Perkin-Elmer 2400 CHN analyser (Waltham, MA, USA) from Universidad Autónoma, Spain.

#### 2.2.3. Gel Permeation Chromatography (GPC)

The molecular weights (*M*_n_) and the molecular mass distributions (*M*_w_/*M*_n_) of polymer samples were measured by Gel Permeation Chromatography (GPC) performed on a Shimadzu LC-20AD GPC (Kyoto, Japan) equipped with a TSK-GEL G3000Hxl column and an ELSD-LTII light-scattering detector. The GPC column was eluted with THF at 40 °C at 1 mL/min and was calibrated using eight monodisperse polystyrene standards in the range 580–483,000 Da. MALDI-ToF MS spectra were acquired with a Bruker Autoflex II ToF/ToF spectrometer (Billerica, MA, USA), using a nitrogen laser source (337 nm, 3 ns) in linear mode with a positive acceleration voltage of 20 kV. Samples were prepared as follows: PC (2 mg) was dissolved in HPLC quality THF with dithranol as matrix and NaOAc as cationization agent in a 100:5:5 ratios. Before evaporation, 10 μL of the mixture solution was deposited on the sample plate. External calibration was performed by using Peptide Calibration Standard II (covered mass range: 700–3200 Da) and Protein Calibration Standard I (covered mass range: 5000–17,500 Da).

#### 2.2.4. Crystallographic Refinement and Structure Solution

Crystals suitable for X-ray diffraction were obtained for **4**, **5** and **6**. The crystals were selected under oil and attached to the tip of a nylon loop. The crystals were mounted in a stream of cold nitrogen at 240–250 K for **4** and **6** and centred in the X-ray beam.

The crystal evaluation and data collection were performed on a Bruker X8 APEX II CCD-based diffractometer with MoKα (λ = 0.71073 Å) radiation. The initial cell constants were obtained from three series of scans at different starting angles. The reflections were successfully indexed by an automated indexing routine built in the SAINT program [54]. The absorption correction was based on fitting a function to the empirical transmission surface as sampled by multiple equivalent measurements [55]. A successful solution by the direct methods [56,57] provided most non-hydrogen atoms from the E-map. The remaining non-hydrogen atoms were located in an alternating series of least-squares cycles and difference Fourier maps. All non-hydrogen atoms were refined with anisotropic displacement coefficients. All hydrogen atoms were included in the structure factor calculation at idealized positions and were allowed to ride on the neighbouring atoms with relative isotropic displacement coefficients. Compounds **4** and **5** show some disordered molecules of THF solvent and we have considered appropriate squeeze them [58].

Final R(F), wR(F2) and goodness-of-fit agreement factors, details on the data collection and analysis can be found in Table S1.

### 2.3. General Procedures

#### 2.3.1. Preparation of Compounds 1−6

**Synthesis of [Zn(2,6-Me_2_C_6_H_3_O)(bpzampe)]_2_ 1.**

In a 250 cm^3^ Schlenk tube, [Zn(Me)(bpzampe)] [29] (1.0 g, 2.31 mmol) was dissolved in dry toluene (60 mL) and the solution was cooled to 0 °C. A solution of 2,6-dimethylphenol (0.28 g, 2.31 mmol) in toluene was added, and the mixture was allowed to warm up to room temperature and stirred during 1 h. The solvent was evaporated to dryness under reduced pressure to yield a white product. The product was washed with *n*-hexane (1 × 25 mL) to give compound **1** as a white solid. Yield: (1.12 g, 90%) Anal. Calcd. For C_56_H_70_N_10_O_4_Zn_2_: C, 62.39; H, 6.55; N, 12.99. Found: C, 62.08; H, 6.58; N, 13.14. ^1^H NMR (C_6_D_6_, 297 K), δ 7.15 (d, ^3^J_H-H_ = 7.5 Hz, 4H, N-Ph^o^), 6.98 (bs, 4H, *m*-H-OAr), 6.83 (t, ^3^J_H-H_ = 7.1 Hz, 2H, *p*-H-OAr), 6.41 (d, ^3^J_H-H_ = 7.5 Hz, 4H, N-Ph^m^), 5.53 (s, 2H, CH^b^), 5.48 (s, 2H, CH^a^), 5.31, 5.10 (s, 4H, H^4,4′^), 2.42 (bs, 12H, (CH_3_)_2_-OAr), 2.38 (s, 12H, -N-(CH_3_)_2_), 2.28 (s, 6H, Me^3^), 2.18 (s, 6H, Me^3′^), 1.79 (s, 6H, Me^5′^), 1.11 (s, 6H, Me^5^). ^13^C {^1^H} NMR (C_6_D_6_, 297 K), δ 161.6 (*ipso*-C-OAr), 159.0-153.0 (C^3,3′^, C^5,5′^), 132.0 (N-Ph^o^), 128.6 (*o*-C-OAr), 127.3 (*m*-C-OAr), 124.1 (*p*-C-OAr), 111.5 (N-Ph^m^), 102.2 (C^4′^), 101.0 (C^4^), 74.9 (C^a^), 72.1 (C^b^), 39.8 (Me_2_-OAr), 25.1 (N-CH_3_), 13.5, 13.3 (Me^3,3′^), 10.2, 9.9 (Me^5,5′^).

**Synthesis of [Zn(2,6-Me_2_C_6_H_3_O)(bpzaepe)]_2_ 2.**

The synthesis of **2** was carried out in an identical manner to **1**, using **[Zn(Me)(bpzaepe)]** (1.0 g, 2.17 mmol) and 2,6-dimethylphenol (0.26 g, 2.17 mmol), to give **2** as a white solid. Yield: (1.17 g, 91%). Anal. Calcd. For C_60_H_78_N_10_O_4_Zn_2_: C, 63.54; H, 6.93; N, 12.35. Found: C, 63.59; H, 6.98; N, 12.43. ^1^H NMR (C_6_D_6_, 297 K), δ 7.17 (d, ^3^J_H-H_ = 7.5 Hz, 4H, N-Ph^o^), 7.04 (bs, 4H, *m*-H-OAr), 6.75 (t, ^3^J_H-H_ = 7.1 Hz, 2H, *p*-H-OAr), 6.59 (d, ^3^J_H-H_= 7.5 Hz, 4H, N-Ph^m^), 5.51 (s, 2H, CH^b^), 5.43 (s, 2H, CH^a^), 5.42, 5.18 (s, 4H, H^4,4′^), 2.95 (m, 8H, N-CH_2_CH_3_), 2.63, 2.30 (bs, 12H, (CH_3_)_2_-OAr), 2.16 (s, 6H, Me^3^), 1.97 (s, 6H, Me^3′^), 1.72 (s, 6H, Me^5′^), 1.12 (s, 6H, Me^5^), 0.89 (t, ^3^J_H-H_ = 8.0 Hz, 12H, N-CH_2_CH_3_). ^13^C {^1^H} NMR (C_6_D_6_, 297 K), δ 158.1 (*ipso*-C-OAr), 156.5-153.9 (C^3,3′^, C^5,5′^), 132.0 (N-Ph^o^), 128.4 (*o*-C-OAr), 127.9 (*m*-C-OAr), 124.2 (*p*-C-OAr), 111.5 (N-Ph^m^), 102.3 (C^4′^), 101.1 (C^4^), 76.9 (C^a^), 71.4 (C^b^), 39.9 (Me_2_-OAr), 39.1 (N-CH_2_CH_3_), 13.4, 13.3 (Me^3,3′^), 12.1 (N-CH_2_CH_3_), 10.2, 9.8 (Me^5,5′^).

**Synthesis of [Zn(bpzampe)_2_{Zn(2,6-Me_2_C_6_H_3_S)_2_}] 3.**

The synthesis of **3** was carried out in an identical manner to **1**, using **[Zn(Me)(bpzampe)]** (1.0 g, 2.31 mmol) and 2,6-dimethylthiophenol (0.31 mL, 2.31 mmol) to give **3** as a pale yellow solid. Yield: (1.21 g, 95%). Anal. Calcd. For C_56_H_70_N_10_O_2_S_2_Zn_2_: C, 60.59; H, 6.36; N, 12.62. Found: C, 60.63; H, 6.40; N, 12.55. ^1^H NMR (C_6_D_6_, 297 K): δ 7.78 (d, ^3^J_H-H_ = 7.5 Hz, 4H, N-Ph^o^), 6.95 (bs, 4H, *m*-H-SAr), 6.81 (t, ^3^J_H-H_ = 7.1 Hz, 2H, *p*-H-SAr), 6.81 (d, ^3^J_H-H_ = 7.5 Hz, 4H, N-Ph^m^), 6.21 (s, 2H, CH^b^), 6.10 (s, 2H, CH^a^) 5.78, 5.26 (s, 4H, H^4,4′^), 2.62 (s, 12H, -N-(CH_3_)_2_), 2.58 (bs, 12H, (CH_3_)_2_-SAr), 2.33 (s, 6H, Me^3^), 1.81 (s, 6H, Me^3′^), 1.63 (s, 6H, Me^5′^), 1.49 (s, 6H, Me^5^). ^13^C {^1^H} NMR (C_6_D_6_, 297 K), δ 158.3 (*ipso*-C-SAr), 142.5-138.0 (C^3,3′^, C^5,5′^), 128.9 (N-Ph^o^), 128.6 (*o*-C-SAr), 128.3 (*m*-C-SAr), 127.5 (*p*-C-SAr), 111.8 (N-Ph^m^), 106.6 (C^4′^), 106.5 (C^4^), 77.6 (C^a^), 70.0 (C^b^), 24.5 (Me_2_-SAr), 19.8 (N-CH_3_), 13.8, 13.7 (Me^3,3′^), 10.2, 10.1 (Me^5,5′^).

**Synthesis of [Zn(bpzaepe)_2_{Zn(2,6-Me_2_C_6_H_3_S)_2_}] 4.**

The synthesis of **4** was carried out in an identical manner to **1**, using **[Zn(Me)(bpzaepe)]** (1.0 g, 2.17 mmol) and 2,6-dimethylthiophenol (0.29 mL, 2.17 mmol), to give **4** as a pale yellow solid. This complex was crystallized in 20 mL of THF and crystals sustainable for X ray diffraction analysis were obtained. Yield: (1.22 g, 97%). Anal. Calcd. For C_60_H_78_N_10_O_2_S_2_Zn_2_: C, 61.79; H, 6.74; N, 12.01. Found: C, 61.89; H, 6.63; N, 12.00. ^1^H NMR (CDCl_3_, 297 K): δ 7.77 (d, ^3^J_H-H_= 7.5 Hz, 4H, N-Ph^o^), 6.98 (bs, 4H, *m*-H-SAr), 6.82 (t, ^3^J_H-H_ = 7.1 Hz, 2H, *p*-H-SAr), 6.81 (d, ^3^J_H-H_ = 7.5 Hz, 4H, N-Ph^m^), 6.21 (s, 2H, CH^b^), 6.11 (s, 2H, CH^a^) 5.79, 5.25 (s, 4H, H^4,4′^), 3.10 (m, 8H, N-CH_2_CH_3_), 2.58 (bs, 12H, (CH_3_)_2_-SAr), 2.15, 1.82 (s, 12H, Me^3,3′^), 1.63 (s, 6H, Me^5′^), 1.50 (s, 6H, Me^5^), 1.00 (t, ^3^J_H-H_ = 8.0 Hz, 12H, N-CH_2_CH_3_). ^13^C {^1^H} NMR (C_6_D_6_, 297 K), δ 159.1 (*ipso*-C-SAr), 142.2-138.1 (C^3,3′^, C^5,5′^), 129.3 (N-Ph^o^), 128.6 (*o*-C-SAr), 128.3 (*m*-C-SAr), 126.3 (*p*-C-SAr), 111.9 (N-Ph^m^), 107.3 (C^4′^), 107.2 (C^4^), 78.0 (C^a^), 70.1 (C^b^), 44.0 (N-CH_2_CH_3_), 24.6 (Me_2_-SAr), 13.7, 13.6 (Me^3,3′^), 12.5 (N-CH_2_CH_3_), 10.2, 10.1 (Me^5,5′^).

**Synthesis of [Zn(2,6-Me_2_C_6_H_3_S)_2_(Hbpzampe)] 5.**

The synthesis of **5** was carried out in an identical manner to **3**, but using two equivalents of 2,6-dimethylthiophenol (0.62 mL, 4.62 mmol), to give **5** as a pale yellow solid. This complex was crystallized in 20 mL of THF and crystals sustainable for X ray diffraction analysis were obtained. Yield: (1.50 g, 94%). Anal. Calcd. For C_36_H_45_N_5_OS_2_Zn: C, 62.37; H, 6.54; N, 10.10. Found: C, 62.47; H, 6.56; N, 10.21. ^1^H NMR (CDCl_3_, 297 K): δ 6.98 (d, ^3^J_H-H_ = 7.5 Hz, 2H, N-Ph^o^), 6.92 (bs, 2H, *m*-H-SAr), 6.83 (t, ^3^J_H-H_ = 7.1 Hz, 1H, *p*-H-SAr), 6.39 (d, ^3^J_H-H_= 7.5 Hz, 2H, N-Ph^m^), 5.61 (s, 1H, CH^b^), 5.51 (s, 1H, CH^a^), 5.40, 5.10 (s, 2H, H^4,4′^), 3.01 (s, 6H, -N-(CH_3_)_2_), 2.41, 2.34 (bs, 12H, (CH_3_)_2_-SAr), 2.25 (s, 3H, Me^3^), 2.18 (s, 3H, Me^3′^), 1.83 (s, 3H, Me^5′^), 1.18 (s, 3H, Me^5^). ^13^C {^1^H} NMR (C_6_D_6_, 297 K), δ 160.3 (*ipso*-C-SAr), 152.3-141.1 (C^3,3′^, C^5,5′^), 129.8 (N-Ph^o^), 129.6 (*o*-C-SAr), 128.3 (*m*-C-SAr), 122.5 (*p*-C-SAr), 111.9 (N-Ph^m^), 107.2 (C^4′^), 106.3 (C^4^), 78.3 (C^a^), 68.6 (C^b^), 24.1, 22.2 (Me_2_-SAr), 19.6 (N-CH_3_), 13.9, 13.8 (Me^3,3′^), 10.2, 9.9 (Me^5,5′^).

**Synthesis of [Zn(2,6-Me_2_C_6_H_3_S)_2_(Hbpzaepe)] 6.**

The synthesis of **6** was carried out in an identical manner to **4**, but using two equivalents of 2,6-dimethylthiophenol (0.58 mL, 4.34 mmol) to give **6** as a pale yellow solid. This complex was crystallized in 20 mL of THF and crystals sustainable for X ray diffraction analysis were obtained. Yield: (1.44 g, 92%). Anal. Calcd. For C_38_H_49_N_5_OS_2_Zn: C, 63.27; H, 6.85; N, 9.71. Found: C, 63.40; H, 6.72; N, 10.03. ^1^H NMR (C_6_D_6_, 297 K), δ 6.97 (d, ^3^J_H-H_ = 7.5 Hz, 2H, N-Ph^o^), 6.95 (bs, 2H, *m*-H-SAr), 6.85 (t, ^3^J_H-H_ = 7.1 Hz, 1H, *p*-H-SAr), 6.37 (d, ^3^J_H-H_ = 7.5 Hz, 2H, N-Ph^m^), 5.58 (s, 1H, CH^b^), 5.45 (s, 1H, CH^a^) 5.39, 5.10 (s, 2H, H^4,4′^), 2.84 (m, 4H, N-CH_2_CH_3_), 3.00, 2.33 (bs, 12H, (CH_3_)_2_-SAr), 2.30, 2.19 (s, 6H, Me^3,3′^), 1.91 (s, 3H, Me^5′^), 1.18 (s, 3H, Me^5^), 0.83 (t, 6H, ^3^J_H-H_ = 8.0 Hz, N-CH_2_CH_3_). ^13^C {^1^H} NMR (C_6_D_6_, 297 K), δ 160.3 (*ipso*-C-SAr), 152.3-141.4 (C^3,3′^, C^5,5′^), 129.9 (N-Ph^o^), 128.6 (*o*-C-SAr), 128.3 (*m*-C-SAr), 122.5 (*p*-C-SAr), 111.8 (N-Ph^m^), 107.2 (C^4′^), 106.3 (C^4^), 78.2 (C^a^), 68.4 (C^b^), 44.0 (N-CH_2_CH_3_), 24.1, 22.5 (Me_2_-SAr), 13.9, 13.8 (Me^3,3′^), 12.1 (N-CH_2_CH_3_), 10.1, 9.9 (Me^5,5′^), 10.0 (N-CH_2_CH_3_).

#### 2.3.2. General Procedure for the Synthesis of Polycarbonates

Cyclohexene oxide (0.98 g, 10.0 mmol) and catalysts **2**, **4** and **6** (0.1 mmol) were placed in a stainless-steel reactor with a magnetic stirrer bar. The autoclave was sealed, pressurized to 5 bar with CO_2_, heated to the desired temperature and then pressurized to 1–40 bar with CO_2_. The reaction mixture was subsequently stirred at 50–100 °C for 2–16 h.

The conversion of cyclohexene oxide into poly(cyclohexene carbonate) was determined by analysis of a sample by ^1^H NMR spectroscopy, and expressed as a percentage of CHO conversion vs the theoretical maximum (100%), determined from the ^1^H NMR spectrum by comparison of the relative integrals of the resonances assigned to the carbonate (4.65 ppm for PCHC and 4.00 ppm for *trans*-cyclic carbonate) and ether (3.45 ppm) linkages, if present, against CHO (3.00 ppm).

The percentage of *trans*-cyclohexene carbonate was determined by analysis of a sample by ^1^H NMR spectroscopy, and expressed as a percentage of *trans*-CHC vs the theoretical maximum (100%), determined from the ^1^H NMR spectrum by comparison of the relative integrals of the resonances assigned to the carbonate (4.00 ppm for *trans*-CHC vs 4.65 ppm for PCHC).

The percentage of poly(cyclohexene carbonate) was determined by analysis of a sample by ^1^H NMR spectroscopy, and expressed as a percentage of PCHC vs the theoretical maximum (100%), determined from the ^1^H NMR spectrum by comparison of the relative integrals of the resonances assigned to the carbonate (4.65 ppm for PCHC vs 4.00 ppm for *trans*-CHC).

The percentage of carbonate linkages was determined by analysis of a sample by ^1^H NMR spectroscopy, and expressed as a percentage of carbonate linkages vs the theoretical maximum (100%), determined from the ^1^H NMR spectrum by comparison of the relative integrals of the resonances assigned to the carbonate (4.65 ppm for PCHC and 4.00 ppm for *trans*-cyclic carbonate) and ether (3.45 ppm) linkages, if present.

The crude product was purified by dissolving it in dichloromethane (10 mL) and by precipitation with the addition of methanol (3 × 100 mL) to form a white solid. The isolated polymer was dried under vacuum at 50 °C for 48 h.

## 3. Results

### 3.1. Synthesis and Characterization of Complexes

**Scheme 2:**
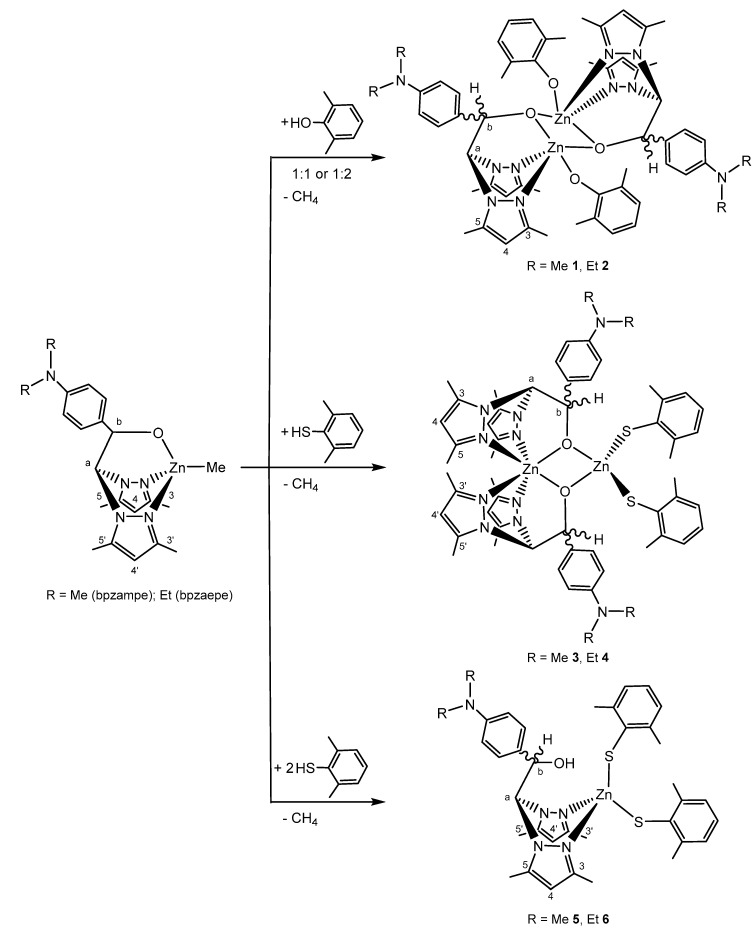
Synthesis of NNO-scorpionate aryloxide and thioaryloxide zinc complexes **1**–**6**.

We explored additional aspects concerning to the reactivity of the previously described in our group mononuclear chiral complexes [Zn(Me)(*κ*^3^-NNO)] [53] [*κ*^3^-NNO = bpzampe = {2,2-bis(3,5-dimethylpyrazol-1-yl)-1-[4-(dimethylamino)phenyl]ethoxide} or bpzaepe = {2,2-bis(3,5-dimethylpyrazol-1-yl)-1-[4-(diethylamino)phenyl]ethoxide}], with several aromatic alcohols or thioalcohols, and new complexes that contain aryloxide or thioaryloxide ligands were isolated after methane elimination. Thus, the alcoholysis or thioalcoholysis reaction of these monoalkyl complexes with ArEH (Ar = 2,6-Me_2_C_6_H_3_; E = O, S), in a 1:1 molar ratio, yields the chiral dinuclear zinc complexes [Zn(OAr)(*κ*^2^-NN*μ*-O)]_2_ **1**–**2** (*κ*^2^-NN*μ*-O = bpzampe **1**, bpzaepe **2**) and [Zn(*κ*^2^-NN-*μ*-O)_2_{Zn(SAr)_2_}] **3**–**4** (*κ*^2^-NN*μ*-O = bpzampe **3**, bpzaepe **4**), whereas when this reaction was carried out in a 1:2 molar ratio of thioalcohol, the mononuclear compounds [Zn(SAr)_2_(*κ*^2^-NN-OH)] **5**–**6** (*κ*^2^-NN-OH = Hbpzampe **5**, Hbpzaepe **6**) were obtained (see Scheme 2). The analogous reaction (1:2 molar ratio) with the alcohol led to dinuclear complexes **1**–**2**.

The different complexes were characterized spectroscopically (see Figures S1–S3). The ^1^H and ^13^C{^1^H} NMR spectra of **1**–**6** exhibit two distinct sets of pyrazole resonances, which indicate the existence of two types of pyrazole rings. The ^1^H NMR spectra of these complexes show two singlets for each of the H^4^, Me^3^ and Me^5^ pyrazole protons, one broad singlet for each of the methine groups (the bridging CH^a^ group to the two pyrazole rings and the sterogenic CH^b^), and the signals corresponding to the R′ moieties of the scorpionate ligands as well as the alkyl, aryloxide or thioaryloxide ligands.

These results are consistent with a geometric environment for the zinc atoms in which the two pyrazole rings are located in *cis* and *trans* positions with respect to the 4-(dimethylamino)phenyl or 4-(diethylamino)phenyl groups (see Scheme 2). The ^1^H NOESY-1D experiments enabled the unequivocal assignment of all ^1^H resonances, and the assignment of the ^13^C {^1^H} NMR signals was carried out on the basis of ^1^H-^13^C heteronuclear correlation (g-HSQC) experiments. In addition, in the dinuclear complexes **1**–**4**, the fact that only two sets of signals are observed for the pyrazole resonances indicates that only one diastereoisomer was present in solution of the two possible (*meso* and *rac*).

The dimeric structure proposed for complexes **1** and **2** (see Scheme 2) is in good agreement with the NMR experiments. The heteroscorpionate ligand is attached to the zinc centre through the nitrogen atoms from both pyrazole rings, and the oxygen atom from the alkoxide moiety bridges the two zinc centres in a *κ*^2^-NN*μ*-O coordination mode. In addition, each zinc atom is coordinated to an aryloxide ligand. The geometry around each zinc metal is a distorted square planar pyramid. This proposed structure is similar to that found for the compound [Zn(OAr)(bpzte)]_2_ (bpzte = 2,2-bis(3,5-dimethylpyrazol-1-yl)-1-para-tolylethoxide], previously reported [48], which was obtained by an analogous reaction. In that complex, X-ray diffraction analysis confirmed this geometrical disposition, with a rhomboidal (ZnO)_2_ core similar to that proposed for complexes **1** and **2**.

**Figure 1:**
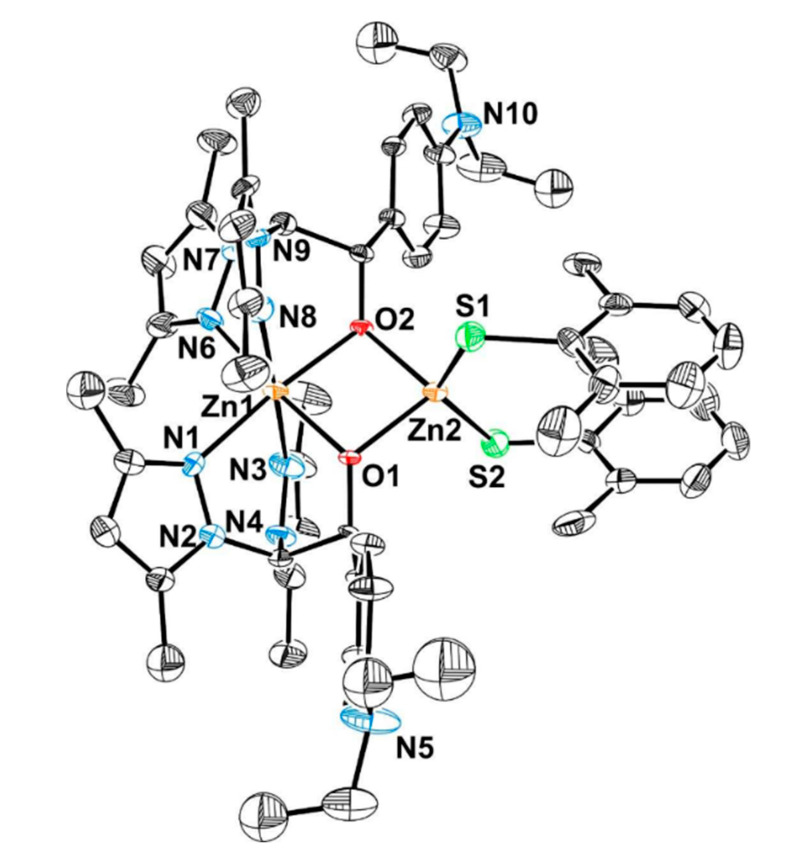
ORTEP view of the *S*,*S* enantiomer of [Zn(bpzaepe)_2_{Zn(2,6-Me_2_C_6_H_3_S)_2_}] (**4**). Hydrogen atoms have been omitted for clarity. Thermal ellipsoids are drawn at the 30% probability level.

An X-ray crystal structure determination was carried out for **4**. The ORTEP drawing is shown in Figure 1. A summary of bond lengths and angles is presented in Table 1 and the crystallographic details are reported in Table S1. Only one diastereoisomer, namely “rac”, was present in the unit cell. These studies confirmed that the presence in solution of only one diastereoisomer for these compounds is maintained in the solid state. The complexes have a dinuclear structure with two μ-bridging alkoxide groups from the scorpionate ligands between the two six- and four-coordinate Zn(II) atoms. The first zinc centre Zn(1) has a distorted octahedral geometry with a heteroscorpionate ligand that acts in a tridentate fashion. The pyrazolic nitrogens N(1), N(3), N(6) and N(8) occupy four positions and the alkoxide oxygen-bridging μ-O(1) and μ-O(2) occupy the other two positions. The second zinc centre Zn(2) has a distorted tetrahedral geometry in which μ-O(1) and μ-O(2) occupy two positions, and the thioaryloxide groups the other two positions [Zn(2)–S distances = 2.287(3) and 2.277(3)]. Furthermore, the X-ray structure of **4** has a rhomboidal (ZnO)_2_ core with Zn(1)–O(1), Zn(1)–O(2), Zn(2)–O(1) and Zn(2)–O(2) bond lengths ranging from 2.032(5) to 2.048(5) Å and the Zn···Zn diagonal 2.974(1) Å is much longer than the O···O diagonal 2.793(5) Å. The dimeric aggregate is based on Zn_2_O_2_ four-membered rings, which have previously been observed in other zinc compounds that contain, for example, thiolate-oxo, alkoxide-imino, aryloxide or aminoalcoholate ligands [59,60,61,62], and, more recently, by our research group with dinuclear complexes of the type [Zn(R)(κ-NNμ-O)]_2_ [48]. However, it should be noted that only one dinuclear compound of zinc with two six- and four-coordinate Zn(II) atoms, based on Zn_2_O_2_ four-membered rings, containing a scorpionate ligand have been reported in the literature [50].

**Figure 2:**
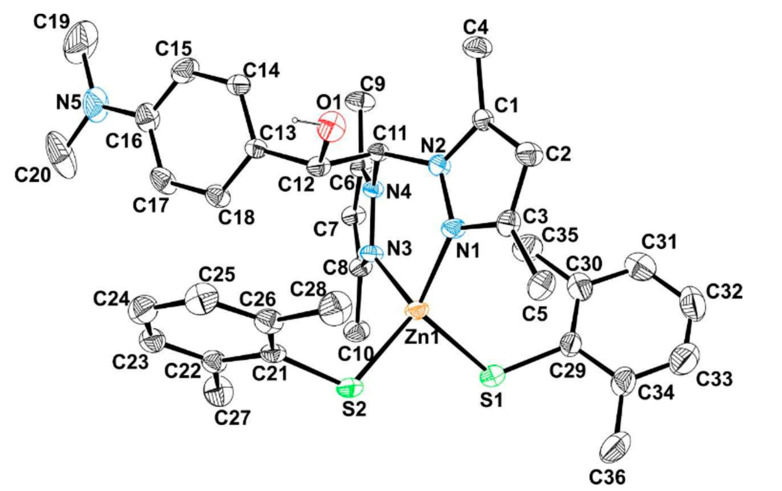
ORTEP view of the *S* enantiomer of [Zn(2,6-Me_2_C_6_H_3_S)_2_(Hbpzampe)] (**5**). Hydrogen atoms have been omitted for clarity. Thermal ellipsoids are drawn at the 30% probability level.

Complexes **5** and **6** were also characterized by single-crystal X-ray diffraction and the molecular structures are shown in Figure 2 and Figure S4, respectively. This study confirmed that the presence in solution of the corresponding two enantiomers (R + S) is maintained in the solid state. The most representative bond lengths and angles, and crystallographic details are presented in Table 1 and Table S1, respectively. These complexes have a monomeric structure that consists of a heteroscorpionate ligand bonded to the zinc atom through the two nitrogen atoms in a κ^2^-NN-coordination mode. In addition, the zinc centre is coordinated to two thioaryloxide ligands. This centre has a distorted tetrahedral environment due to the κ^2^-NN-coordination of the scorpionate ligand with major distortion in the N(1)–Zn(1)–N(3) angle, which has a value of 91.6(2)° for complex **5**. The Zn–N distances [2.047(5) Å and 2.067(5) Å] for Zn(1)–N(1) and Zn(1)–N(3) in complex **5**, are in good agreement with other values determined for zinc scorpionate complexes such as [Zn(CH_3_)(bpzbe)] [63] or [Zn(CH_3_)(pbp^t^amd)] [64] prepared by our research group. Finally, the Zn–S distances [2.290(2) Å and 2.265(2) Å] for **5** are similar to those found for complex **4**.

### 3.2. Catalytic Studies on the Ring-Opening Copolymerization of Cyclohexene Oxide with Carbon Dioxide

**Scheme 3:**
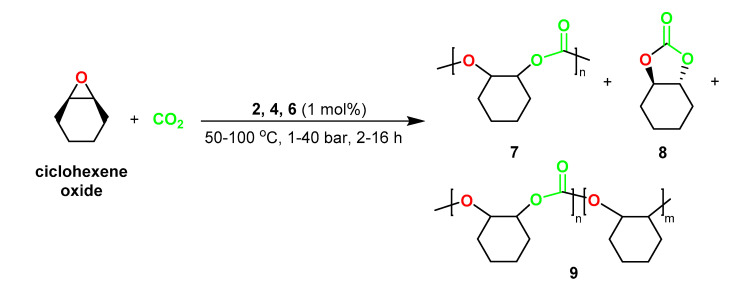
Synthesis of poly(cyclohexene carbonate) catalysed by complexes **2**, **4** and **6**.

A representative complex of each type of molecular arrangement was selected for catalytic inspection. Thus, the dinuclear complexes **2** and **4**, and the mononuclear **6** were initially assessed for the conversion of cyclohexene oxide (CHO) into poly(cyclohexene carbonate (PCHC; **7**) at 80 °C and 40 bar of carbon dioxide pressure in the absence of a co-catalyst under solvent-free conditions for 16 h, using 1 mol% of complexes **2**, **4** and **6** (see Scheme 3).

^1^H NMR spectroscopy was employed to analyse each reaction without further purification and to determine the conversion of CHO into **7**, cyclohexene carbonate (CHC; **8**) or polyether–polycarbonate **9** (see Scheme 3). The results are presented in Table 2. Interestingly, complexes **2**, **4** and **6**, showed good to excellent conversions of cyclohexene oxide, in the absence of a co-catalyst, thus indicating that these complexes are able to initiate copolymerization by themselves [14].

In the case of the dinuclear thioalkoxide derivative **4**, the only polymeric species identified by ^1^H NMR spectroscopy was poly(cyclohexene carbonate) **7**, in conjunction with *trans*-cyclohexene carbonate **8**, as a result of backbiting reactions (Table 2, entry 2). Contrarily, the mononuclear thioalkoxide complex **6** displayed lower selectivity for the formation of **7** (Table 2, entry 3), while the dinuclear alkoxide complex **2** showed a very low selectivity (Table 2, entry 1). Both complexes **2** and **6** presented higher selectivity for polyether polycarbonate **9** production than complex **4** (Table 2, entries 1–3). Thus, complex **4** exhibited outstanding catalytic activity and carbonate selectivity for the ring-opening copolymerization of CHO and carbon dioxide (Table 2, entry 2), affording 75% conversion with a high content of carbonate linkages (>99%) and a very high PCHC/CHC ratio (93/7), possibly due to the presence of the hemilabile thioalkoxide ligand. Interestingly, very efficient and selective multinuclear zinc catalysts bearing different auxiliary ligands have recently been reported in the ROCOP of CHO and CO_2_ (see Chart 1) [17,18,23,24,25,51]. However, as far as we are aware, no examples have been reported containing thioalkoxide auxiliary ligands [65].

Since complex **4** was more active and selective than **2** and **6** for the synthesis of **7**, complex **4** was finally selected for further optimization of the reaction. Thus, the effect of varying catalyst loading, the reaction temperature, reaction pressure and reaction time was inspected. The results are shown in Table S2 and Table 3, Table 4 and Table 5, respectively. Under the previous reaction conditions (80 °C, 40 bar of CO_2_ pressure), the catalyst loading was finally optimized to 1 mol % (Table S2).

In addition, the catalytic activity of complex **4** was very dependent on the reaction temperature (Table 3). Thus, reduction of the temperature from 80 to 50 °C resulted in a dramatic decreasing on both conversion and selectivity of CHO to **7** (52% conv.; PCHC/CHC = 73/27) (Table 3, entry 1).

In addition, decreasing in only 10 °C on the reaction temperature afforded a slight increase in the conversion of CHO into PCHC (79%), which led to the maintenance of selectivity at a PCHC/CHC ratio of 92/8. Conversely, the increasing of the reaction temperature up to 100 °C did not significantly increase the conversion of CHO into **7**, but led to an important decrease in selectivity (PCHC/CHC = 72/28, Table 3, entries 3–5), as a result of the thermodynamic control in the reaction, where CHC formation is favoured. Therefore, the optimized temperature for further experiments was 70 °C.

The reaction pressure was also found to have a marked effect on the catalytic activity and selectivity, as presented on Table 4. Detrimental effect on conversion of complex **4** was observed when decreasing the reaction pressure from 40 to 1 bar (Table 4, entry 1). Interestingly, at 10 bar of CO_2_ pressure, conversion and selectivity values were very similar to that found at 20 bar (72%, PCHC/CHC = 95/5), with the TOF 4.27 h^−1^. Not expectedly, increasing of pressure at 30 bar did not produce a significant growth on the conversion value, while a lack of PCHC/CHC selectivity was observed (83/17). It was also noteworthy that the carbonate linkage (>99%) remained essentially constant in the range of 1 to 40 bar (Table 4, entries 1–5). Therefore, 10 bar was the optimal CO_2_ pressure.

**Figure 3:**
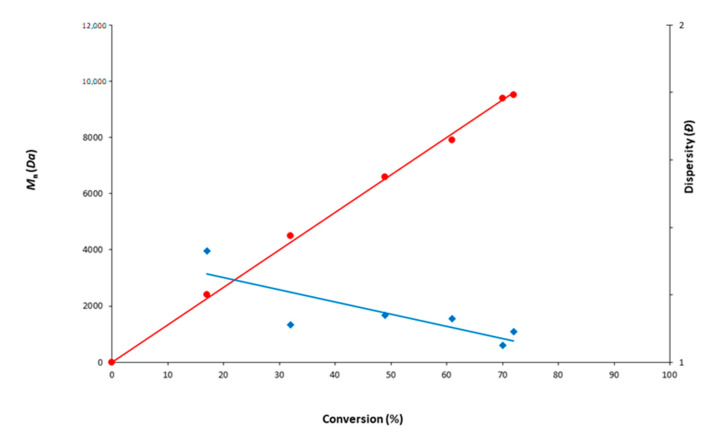
Plot of *M*_n_ versus CHO conversion (●) and *M*_w_/*M*_n_ versus CHO conversion (♦) for compound **4** at 70 °C and 10 bar CO_2_.

Finally, the influence of reaction time on catalytic activity of complex **4** was investigated. As expected, at short periods of time (2 h), the conversion decreased from 72% to 17% (Table 5, entries 1–6), with a slight decreased of polymer selectivity (95–84%). Importantly, increase of the reaction time produced PCHC with higher molecular weights, with low dispersity (*Đ)* values ranging from 1.33–1.05 (see Figure S5), suggesting the absence of back-biting reactions and that the ROCOP of cyclohexene oxide and CO_2_ exhibits pseudo-living propagations (see Figure 3).

Although the activity values found for complex **4** are considerably lower than that reported for alternative very efficient zinc complexes [18,23,24,25], the reaction conditions optimized for **4** are significantly milder than those described for some of these catalysts, as well as analogous previously reported scorpionate zinc complexes [51], showing a high level of PCHC selectivity (see Chart 1). In addition, pseudo-first order with respect to cyclohexene oxide consumption was confirmed from the semi-logarithmic plot of ln[CHO] versus reaction time, which displays a linear increase in monomer conversion with reaction time (see Figure S6).

#### Polymer Microstructure and End-Group Analysis of Poly(Cyclohexene Carbonate) Produced by Complex **4**

**Figure 4:**
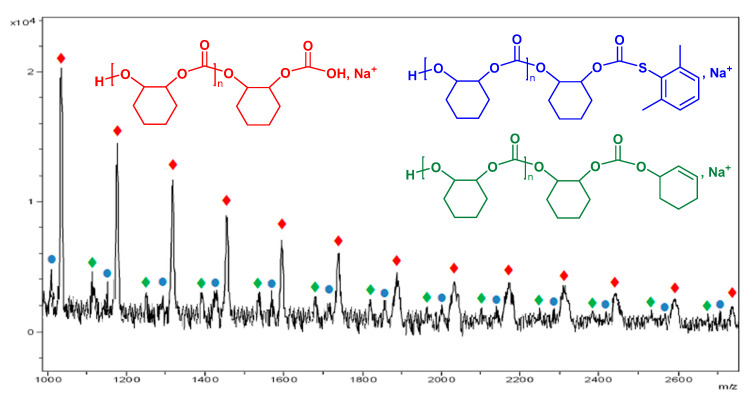
MALDI-ToF mass spectrum of poly(cyclohexene carbonate) sample from Table 4 (entry 2) using zinc complex **4** as catalyst at 70 °C and 10 bar CO_2_. Series ♦ has a repeat unit *m*/*z* = 1+ (142.07 × *DP*_n+1_) + 17 + 22.98, where *n* = 6–18. Series ● has a repeat unit *m*/*z* = 1+ (142.07 × *DP*_n+1_) + 137.14 + 22.98, where *n* = 5–17. Series ♦ has a repeat unit *m*/*z* = 1+ (142.07 × *DP*_n+1_) + 97.05 + 22.98, where *n* = 6–17.

The polymer microstructure was analysed by ^1^H and ^13^C NMR spectroscopy (see Figure S7), and MALDI-TOF MS (matrix assisted laser desorption time-of-flight mass spectrometry) to evaluate the end-group in the polymers (see Figure 4). Thus, tacticity of the materials was examined by ^13^C NMR spectroscopy through the analysis of carbonyl region, which resulted atactic polymers since isotactic (153.7 ppm) and syndiotactic (153.7–153.0 ppm) diads were identified (see Figure S7b).

In addition, MALDI-TOF MS spectrum were acquired employing dithranol as matrix and NaOAc as cationization agent. A PCHC d3.2.istribution including three end-group series of peaks separated by a molecular mass of 142 Da was identified in the spectrum, indicating different initiation possibilities by complex **4**, with the loss of a cyclohexene carbonate unit (C_7_H_10_O_3_) in all polymer chains (see Figure 4). Particularly, it was established a major series possessing two hydroxyl end groups in the polycarbonate (♦), another series bearing one thioaryloxide group and one hydroxyl end-group (●) and a third series containing one cyclohexenyl group and one hydroxyl end-group (♦), which suggest that chain transfer reactions take place due to the existence of either adventitious water and/or cyclohexenediol (produced by the hydrolysis of the CHO) through the polymerization event. This behaviour has also been previously observed [51,66,67].

## 4. Conclusions

In summary, we prepared a series of mono- and dinuclear chiral alkoxide/thioalkoxide scorpionate complexes based on an inexpensive and non-toxic metal. These families of complexes present an interesting variety of structural arrangements, with the scorpionate ligands in different types of coordination modes, which were unambiguously elucidated by X-ray diffraction studies.

These complexes can act as efficient catalysts for the synthesis of poly(cyclohexene carbonate) via ring-opening copolymerization of cyclohexene oxide and carbon dioxide in the absence of a co-catalyst. More interestingly, catalytic efficiency is highly dependent on catalyst nuclearity and substituents. Thus, this new approach has led to the development of an interesting dinuclear thioalkoxide zinc scorpionate [Zn(bpzaepe)_2_{Zn(SAr)_2_}] (**4**) that behaves as an effective and selective initiator for poly(cyclohexene carbonate) production under milder conditions. Thus, catalyst **4** shows high catalytic activity (72% conversion and TOF up to 4.3 h^−^^1^), carbonate linkage (>99%) and polycarbonate selectivity (95%), at 70 °C, 10 bar of CO_2_ pressure after 16 h, under solvent-free conditions, which constitutes a further step forward in the development of inexpensive, more efficient and non-toxic metal-based catalysts for the CO_2_ fixation into the selective production of poly(cyclohexene carbonate) with narrow dispersities [17,18,19,20,21,22,23,24,25,51].

## Figures and Tables

**Table 1:** Selected bond lengths (Å) and angles (°) for **4**, **5** and **6**.

**Bond Lengths**					
**4**		**5**		**6**	
N(1)-Zn(1)	2.133(7)	Zn(1)-N(1)	2.047(5)	Zn(1)-N(1)	2.047(3)
N(3)-Zn(1)	2.230(7)	Zn(1)-N(3)	2.067(5)	Zn(1)-N(3)	2.082(3)
N(6)-Zn(1)	2.117(7)	Zn(1)-S(2)	2.265(2)	Zn(1)-S(1)	2.268(1)
N(8)-Zn(1)	2.226(7)	Zn(1)-S(1)	2.290(2)	Zn(1)-S(2)	2.296(1)
O(1)-Zn(2)	2.032(5)	S(1)-C(29)	1.763(8)	S(1)-C(23)	1.769(5)
O(1)-Zn(1)	2.044(5)	S(2)-C(21)	1.765(7)	S(2)-C(31)	1.771(5)
O(2)-Zn(2)	2.036(4)	O(1)-C(12)	1.414(7)	O(1)-C(12)	1.414(4)
O(2)-Zn(1)	2.048(5)				
S(1)-Zn(2)	2.287(3)				
S(2)-Zn(2)	2.277(3)				
Zn(1)-Zn(2)	2.974(1)				
C(45)-S(1)	1.789(9)				
C(53)-S(2)	1.763(9)				
**Angles**					
S(2)-Zn(2)-S(1)	134.15(9)	S(2)-Zn(1)-S(1)	99.87(8)	S(1)-Zn(1)-S(2)	98.87(4)
O(1)-Zn(1)-O(2)	86.1(2)	N(1)-Zn(1)-N(3)	91.6(2)	N(1)-Zn(1)-N(3)	91.7(1)
N(6)-Zn(1)-N(1)	98.7(3)	N(1)-Zn(1)-S(1)	115.1(2)	N(1)-Zn(1)-S(1)	122.9(1)
N(8)-Zn(1)-N(3)	176.9(3)	N(3)-Zn(1)-S(2)	114.3(2)	N(3)-Zn(1)-S(2)	113.6(1)
O(1)-Zn(2)-O(2)	86.7(2)				

**Table 2:** Synthesis of poly(cyclohexene carbonate) catalysed by complexes **2**, **4** and **6** *^a^*.

Entry	Cat	Conv. (%) *^b^*	%CHC *^b^*	%Copolymer (%Carbonate Linkage) *^b^*	TON *^c^*	TOF *^d^* (h^−1^)
1	**2**	52	44	56(64)	29	1.82
2	**4**	75	7	93(>99)	70	4.36
3	**6**	65	15	85(64)	55	3.45

*^a^* Reactions carried out at 80 °C and 40 bar CO_2_ pressure for 16 h using 1 mol % of catalyst. *^b^* Conversion, % of *trans*-CHC, % of PCHC and % of carbonate linkages determined by ^1^H NMR spectroscopy of the crude reaction mixture (see Section 2.3.2). *^c^* TON = moles of PCHC/moles of catalyst. *^d^* TOF = TON/time (h).

**Table 3:** Effect of reaction temperature on the synthesis of poly(cyclohexene carbonate) catalysed by complex **4** *^a^*.

Entry	Temp (°C)	Conv. (%) *^b^*	%CHC *^b^*	%Copolymer (%Carbonate Linkage) *^b^*	TON *^c^*	TOF *^d^* (h^−1^)	*M*_n(exp)_(*Da*) *^e^* (*M*_w_/*M*_n_) *^e^*
1	50	52	27	73(>99)	38	2.37	5100(1.33)
2	60	62	11	89(>99)	55	3.45	7700(1.27)
3	70	79	8	92(>99)	73	4.54	10,700(1.03)
4	80	75	7	93(>99)	70	4.36	10,100(1.07)
5	100	80	28	72(>99)	58	3.60	8500(1.15)

*^a^* Reactions carried out at 50–100 °C and 40 bar CO_2_ pressure for 16 h using 1 mol % of catalyst **4**. *^b^* Conversion, % of *trans*-CHC, % of PCHC and % of carbonate linkages determined by ^1^H NMR spectroscopy of the crude reaction mixture (see Section 2.3.2). *^c^* TON = moles of product/moles of catalyst. *^d^* TOF = TON/time (h). *^e^* Determined by GPC relative to polystyrene standards in tetrahydrofuran.

**Table 4:** Effect of reaction pressure on the synthesis of poly(cyclohexene carbonate) catalysed by complex **4** *^a^*.

Entry	Pres. (bar)	Conv. (%) *^b^*	%CHC *^b^*	%Copolymer (%Carbonate Linkage) *^b^*	TON *^c^*	TOF *^d^* (h^−1^)	*M*_n(exp)_(*Da*) *^e^* (*M*_w_/*M*_n_) *^e^*
1	1	55	10	90(>99)	50	3.09	7400(1.13)
2	10	72	5	95(>99)	68	4.27	9500(1.09)
3	20	70	3	97(>99)	68	4.24	9800(1.03)
4	30	83	17	83(>99)	69	4.30	10,000(1.07)
5	40	79	8	92(>99)	73	4.54	10,700(1.03)

*^a^* Reactions carried out at 70 °C and 1–40 bar CO_2_ pressure for 16 h using 1 mol% of catalyst **4**. *^b^* Conversion, % of *trans*-CHC, % of PCHC and % of carbonate linkages determined by ^1^H NMR spectroscopy of the crude reaction mixture (see Section 2.3.2). *^c^* TON = moles of product/moles of catalyst. *^d^* TOF = TON/time (h). *^e^* Determined by GPC relative to polystyrene standards in tetrahydrofuran.

**Table 5:** Effect of reaction time on the synthesis of poly(cyclohexene carbonate) catalysed by complex **4** *^a^*.

Entry	Time (h)	Conv. (%) *^b^*	%CHC *^b^*	%Copolymer (%Carbonate Linkage) *^b^*	TON *^c^*	TOF *^d^* (h^−1^)	*M*_n(exp)_(*Da*) *^e^* (*M*_w_/*M*_n_) *^e^*
1	2	17	16	84(>99)	14	7.14	2400(1.33)
2	5	32	14	86(>99)	28	5.50	4500(1.11)
3	8	49	9	91(>99)	45	5.57	6600(1.14)
4	10	61	12	88(>99)	54	5.37	7900(1.13)
5	15	70	7	93(>99)	65	4.34	9400(1.05)
6	16	72	5	95(>99)	68	4.27	9500(1.09)

*^a^* Reactions carried out at 70 °C and 10 bar CO_2_ pressure for 2–16 h using 1 mol% of catalyst **4**. *^b^* Conversion, % of trans-CHC, % of PCHC and % of carbonate linkages determined by ^1^H NMR spectroscopy of the crude reaction mixture (see Section 2.3.2). *^c^* TON = moles of product/moles of catalyst. *^d^* TOF = TON/time (h). *^e^* Determined by GPC relative to polystyrene standards in tetrahydrofuran.

## Supplementary Materials

The following are available online at https://www.mdpi.com/2073-4360/12/9/2148/s1, Figures S1–S3: ^1^H and ^13^C-^1^H NMR spectra of complexes **2**, **4** and **6**, Figure S4: ORTEP view of the complex [Zn(2,6-Me_2_C_6_H_3_S)_2_(bpzaepeH)] **6**, Table S1: Crystal data and structure refinement for **4**, **5** and **6**, Table S2: Effect of catalyst loading on the synthesis of poly(cyclohexene carbonate) catalysed by complex **4**, Figure S5: GPC trace of poly(cyclohexene carbonate) produced by complex **4** at 70 °C and 10 bar CO_2_, Figure S6: Kinetic plot for ring-opening copolymerisation of cyclohexene oxide and carbon dioxide catalysed by complex **4** at 70 °C and 10 bar CO_2_, Figure S7: ^1^H NMR and ^13^C-{^1^H} NMR spectra of poly(cyclohexene carbonate) sample prepared using complex **4** at 70 °C and 10 bar CO_2_.
