# Supplementary material for: Efficient Production of Poly(Cyclohexene Carbonate) via ROCOP of Cyclohexene Oxide and CO2 Mediated by NNO-Scorpionate Zinc Complexes
Source: Polymers (Basel). 2020 Sep 21;12(9):2148. doi: 10.3390/polym12092148 (PMC7569798; doi:10.3390/polym12092148)
Supplement: Supplementary file 1 [file polymers-12-02148-s001.pdf]

Supporting Information for

# Efficient Production of Poly(Cyclohexene Carbonate) via ROCOP of Cyclohexene Oxide and $CO_2$ Mediated by NNO-Scorpionate Zinc Complexes

**Sonia Sobrino [1], Marta Navarro [2], Juan Fernández-Baeza [1], Luis F. Sánchez-Barba [2,*], Agustín Lara-Sánchez [1], Andrés Garcés [2], José A. Castro-Osma [1] and Ana M. Rodríguez [1]**

[1] Centro de Innovación en Química Avanzada (ORFEO-CINQA), Departamento de Química Inorgánica, Orgánica y Bioquímica, Universidad de Castilla-La Mancha, Campus Universitario, 13071 Ciudad Real, Spain; Sonia.Sobrino@uclm.es (S.S.); Juan.FBaeza@uclm.es (J.F.-B.); Agustin.Lara@uclm.es (A.L.-S.); JoseAntonio.Castro@uclm.es (J.A.C.-O.); AnaMaria.RFdez@uclm.es (A.M.R.)

[2] Departamento de Biología y Geología, Física y Química Inorgánica, Universidad Rey Juan Carlos, Móstoles, 28933 Madrid, Spain; marta.navarro.sanz@urjc.es (M.N.); andres.garces@urjc.es (A.G.)

# Table of Contents


**1) Spectroscopy details**

**Figures S1–S3.** $^{1}H$ and $^{13}C$-$^{1}H$ NMR spectra of complexes **2**, **4** and **6** ..................................... S3

**2) X-Ray Diffraction Studies:** Crystallographic structure determination for the complexes **4**, **5** and **6**.

**Figure S4.** ORTEP view of the complex [Zn(2,6-$Me_2C_6H_3S)_2$(bpzaepeH)] **6** ........................... S6

**Table S1**. Crystal data and structure refinement for **4**, **5** and **6** ................................................ S7

**3) Experimental details for the synthesis of poly(cyclohexene carbonate)**

**Table S2**. Effect of catalyst loading on the synthesis of poly(cyclohexene carbonate) catalysed by complex **4** ........................................................................................................................ S7

**Figure S5.** GPC trace of poly(cyclohexene carbonate) produced by complex **4** at 70 °C and 10 bar $CO_2$ ........................................................................................................................ S9

**Figure S6.** Kinetic plot for ring-opening copolymerisation of cyclohexene oxide and carbon dioxide catalysed by complex **4** at 70 °C and 10 bar $CO_2$ ........................................................ S10

**Figure S7.** $^{1}H$ NMR and $^{13}C$-{$^{1}H$} NMR spectra of poly(cyclohexene carbonate) sample prepared using complex **4** at 70 °C and 10 bar $CO_2$ ................................................................ S11

## 1. Spectroscopic Details

**Figures S1–S3.** $^1$H NMR spectrum of complexes **2**, **4** and **6**.

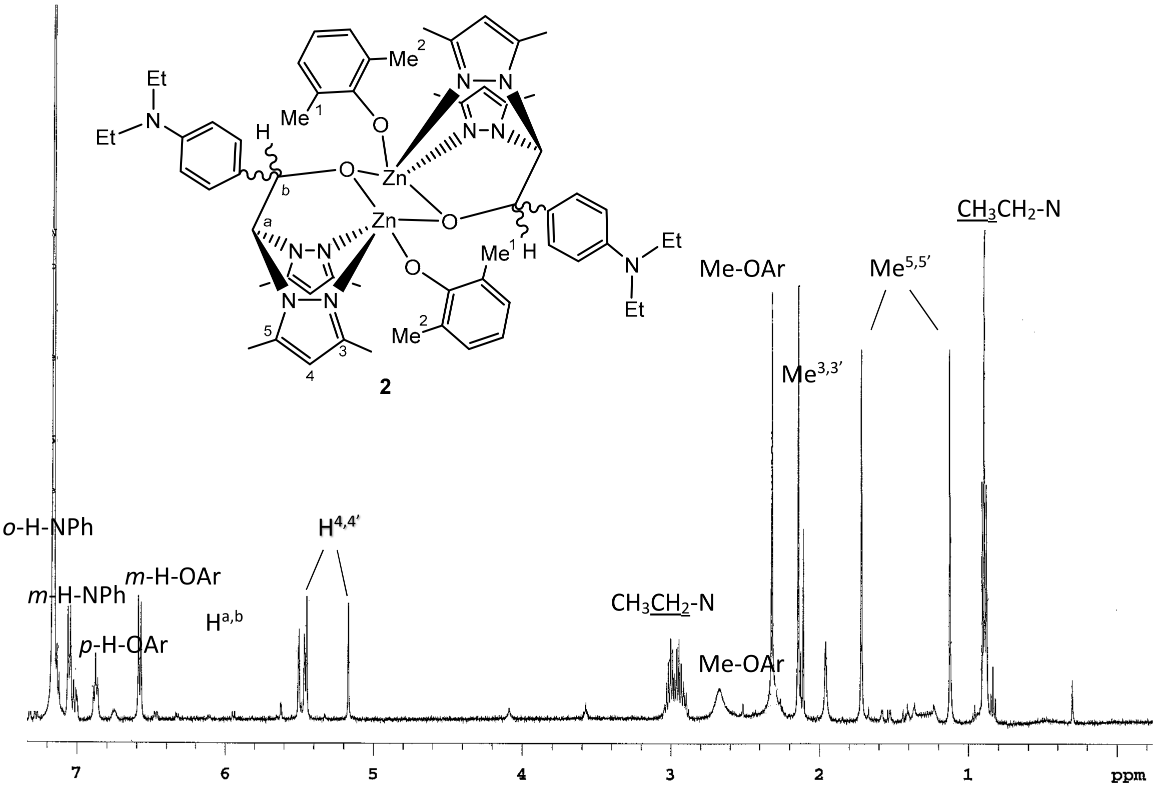


**Figure S1.** $^1$H-NMR spectrum (500 MHz, 297 K, $C_6D_6$) for complex [Zn(2,6-$Me_2C_6H_3O$)(bpzaepe)]$_2$ **2**.

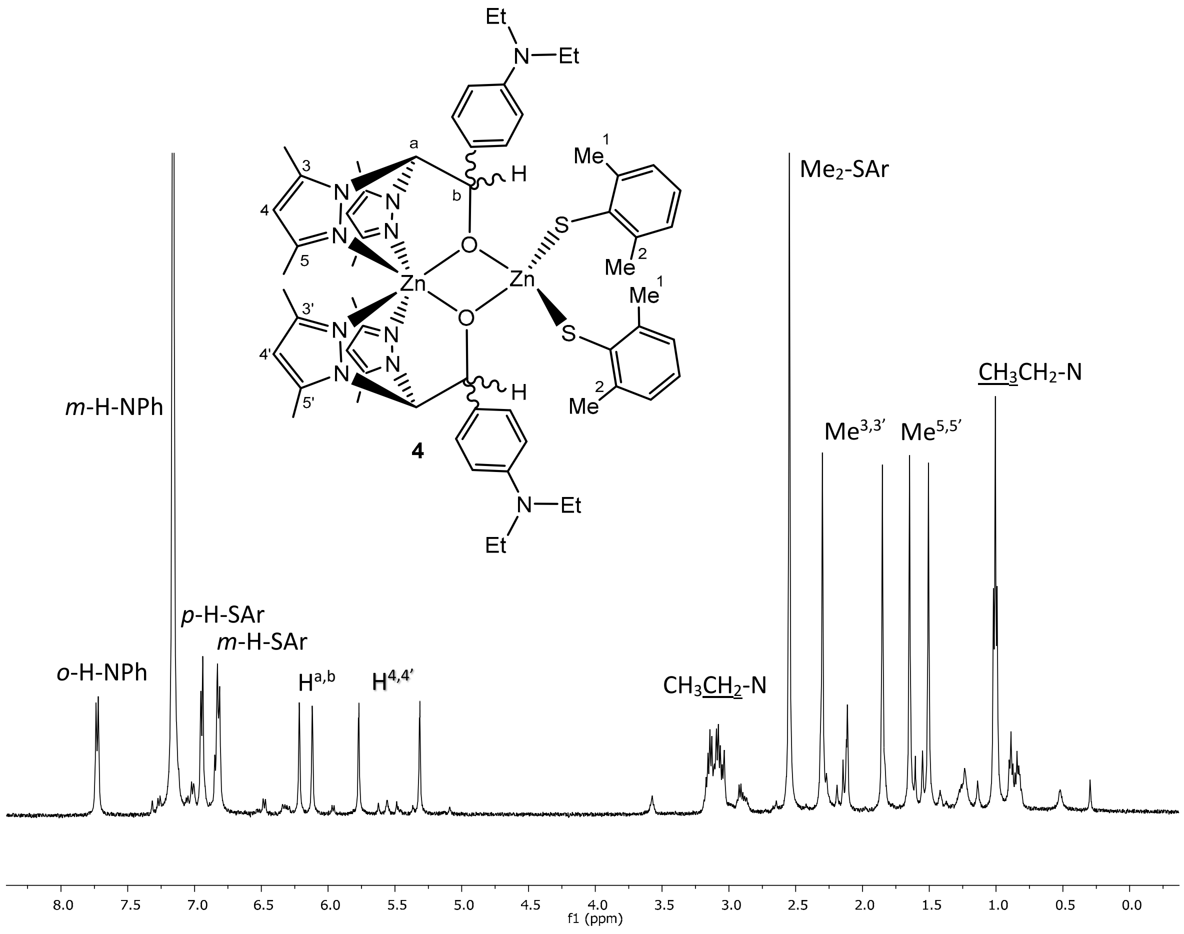


**Figure S2.** $^{1}$H-NMR spectrum (500 MHz, 297 K, $C_6D_6$) for complex [Zn(bpzaepe)$_2${Zn(2,6-$Me_2C_6H_3S$)$_2$}] **4**.

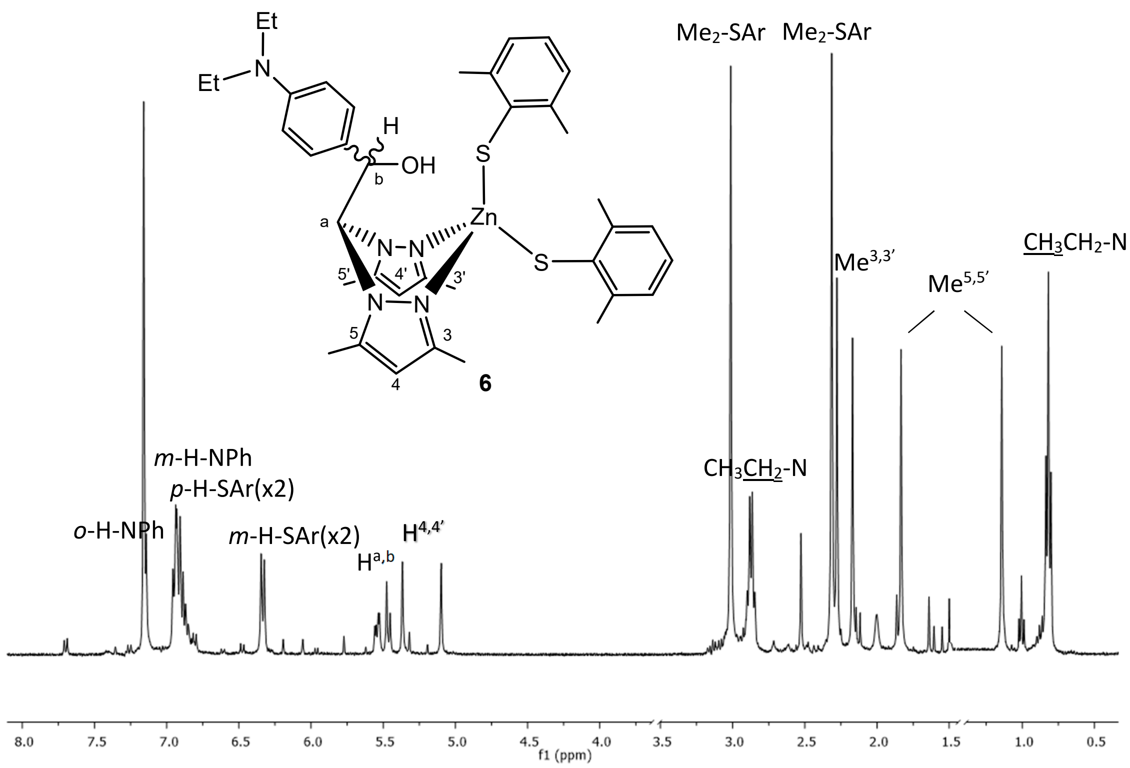


**Figure S3.** $^1$HNMR spectrum (500 MHz, 297 K, $C_6D_6$) for complex [Zn(2,6-$Me_2C_6H_3S)_2$(Hbpzaepe)] **6**.

2. **X-Ray Diffraction Studies:** Crystallographic structure determination for the complexes **4**, **5** and **6**.

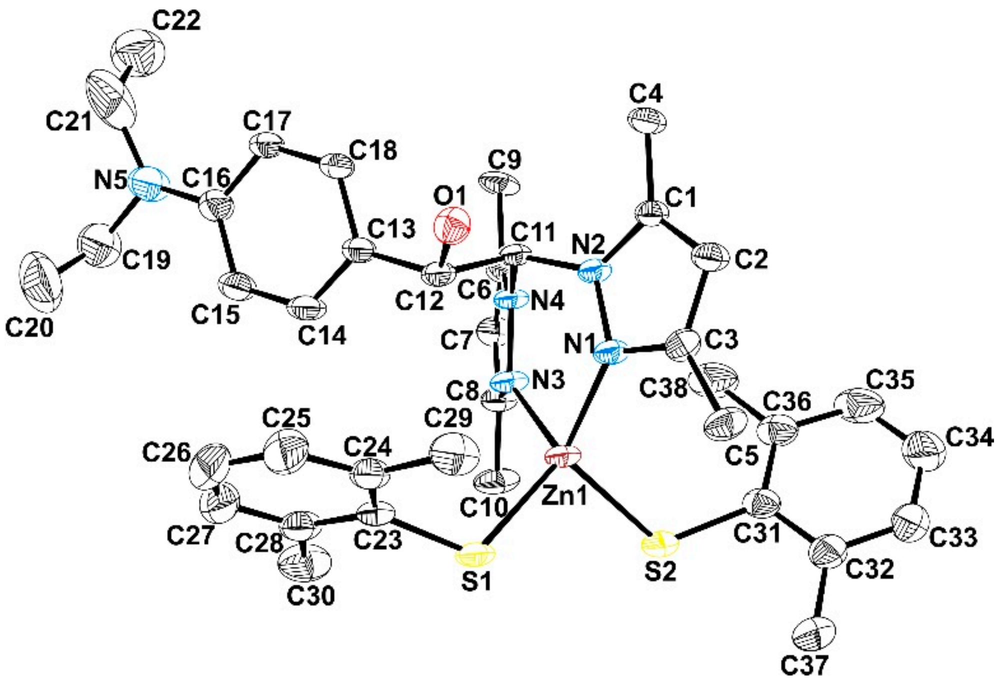


**Figure S4**. ORTEP view of the complex [Zn(2,6-$Me_2C_6H_3S)_2$(bpzaepeH)] **6** Hydrogen atoms have been omitted for clarity. Thermal ellipsoids are drawn at the 30% probability level.

**Table S1**. Crystal data and structure refinement for **4**, **5** and **6**.

| | **4** | **5** | **6** |
|---|---|---|---|
| Empirical formula | $C_{62}H_{82}N_{10}O_{2.5}S_2Zn_2$ | $C_{40}H_{53}N_5O_2S_2Zn$ | $C_{42}H_{57}N_5O_2S_2Zn$ |
| Formula weight | 1202.23 | 765.36 | 793.41 |
| Temperature (K) | 240(2) | 290(2) | 200(2) |
| Wavelength (Å) | 0.71073 | 0.71073 | 0.71073 |
| Crystal system | Triclinic | Monoclinic | Monoclinic |
| Space group | P $\bar{1}$ | P $2_1$/n | C 2/c |
| a(Å) | 14.9313(12) | 16.608(5) | 30.2612(7) |
| b(Å) | 14.9352(13) | 17.144(6) | 16.7513(3) |
| c(Å) | 20.0887(17) | 17.136(6) | 18.4092(3) |
| α(°) | 85.716(4) | 90 | 90 |
| β(°) | 69.721(4) | 113.998(5) | 92.921(2) |
| γ(°) | 76.817(4) | 90 | 90 |
| Volume(Å$^3$) | 4091.4(6) | 4458(3) | 9319.8(3) |
| Z | 2 | 4 | 8 |
| Density (calculated) (g/cm$^3$) | 0.976 | 1.140 | 1.131 |
| Absorption coefficient (mm$^{-1}$) | 0.676 | 0.680 | 0.653 |
| F(000) | 1272 | 1624 | 3376 |
| Crystal size (mm$^3$) | 0.37 x 0.08 x 0.04 | 0.22 x 0.14 x 0.04 | 0.24 x 0.09 x 0.04 |
| Index ranges | $-18 \leq h \leq 17$<br>$-18 \leq k \leq 18$<br>$-24 \leq l \leq 24$ | $-19 \leq h \leq 17$<br>$-20 \leq k \leq 20$<br>$-20 \leq l \leq 20$ | $-35 \leq h \leq 35$<br>$-19 \leq k \leq 19$<br>$0 \leq l \leq 21$ |
| Reflections collected | 86477 | 29051 | 16073 |
| Independent reflections | 14449<br>[R(int) = 0.1416] | 7761<br>[R(int) = 0.1666] | 8203<br>[R(int) = 0.0287] |
| Data / restraints / parameters | 14449 / 5 / 720 | 7761 / 0 / 463 | 8203 / 101 / 588 |
| Goodness-of-fit on F$^2$ | 1.070 | 0.930 | 1.076 |
| Final R indices [$I$>2$\sigma(I)$] | R1 = 0.1007<br>wR2 = 0.2765 | R1 = 0.0794<br>wR2 = 0.1561 | R1 = 0.0667<br>wR2 = 0.1939 |
| Largest diff. peak / hole, e.Å$^{-3}$ | 0.972 and -1.387 | 0.441 and -0.318 | 0.890 d -0.276 |

## 3. Experimental details for the synthesis of poly(cyclohexene carbonate)

**Table S2**. Effect of catalyst loading on the synthesis of poly(cyclohexene carbonate) catalysed by complex **4**[a].

| Entry | 4 (%) | Conv. PCHC (%)[a] | Selectivity PCHC (%)[a] |
|---|---|---|---|
| 1 | 0.5 | 15 | 63 |
| 2 | 0.8 | 50 | 90 |
| 3 | 1.0 | 85 | 93 |
| 4 | 1.25 | 91 | 94 |

[a] Reaction condition: 80 ºC, 40 bar $CO_2$, substrate: cyclohexene oxide, 16 h.

## ==== Shimadzu LCsolution Analysis Report ====

C:\LabSolutions\Data\gpcresultados\2019\8.lcd

| | |
|---|---|
| Acquired by | : Admin |
| Sample Name | : 19 |
| Sample ID | : 10 |
| Vail # | : |
| Injection Volume | : 20 uL |
| Data File Name | : 8.lcd |
| Method File Name | : Metodo bueno 2019.OK.lcm |
| Batch File Name | : SingleRun120120905170518.lcb |
| Report File Name | : Default.lcr |
| Data Acquired | : 05/09/2019 16:05:26 |
| Data Processed | : 05/09/2019 16:24:13 |

**<Chromatogram>**

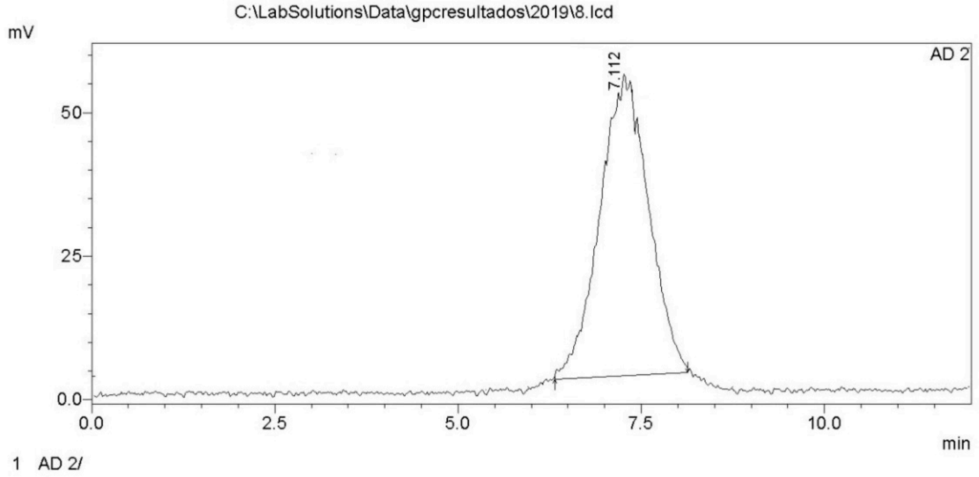


GPC Results

[Average Molecular Weight]

| | |
|---|---|
| Number Average Molecular Weight(Mn) | 9522 |
| Weight Average Molecular Weight(Mw) | 10379 |
| Viscosity Average Molecular Weight(Mv) | 0 |
| Mw/Mn | 1.09 |

**Figure S5.** GPC trace of poly(cyclohexene carbonate) produced by complex **4** at 70 °C and 10 bar $CO_2$ (Table 5, entry 6).

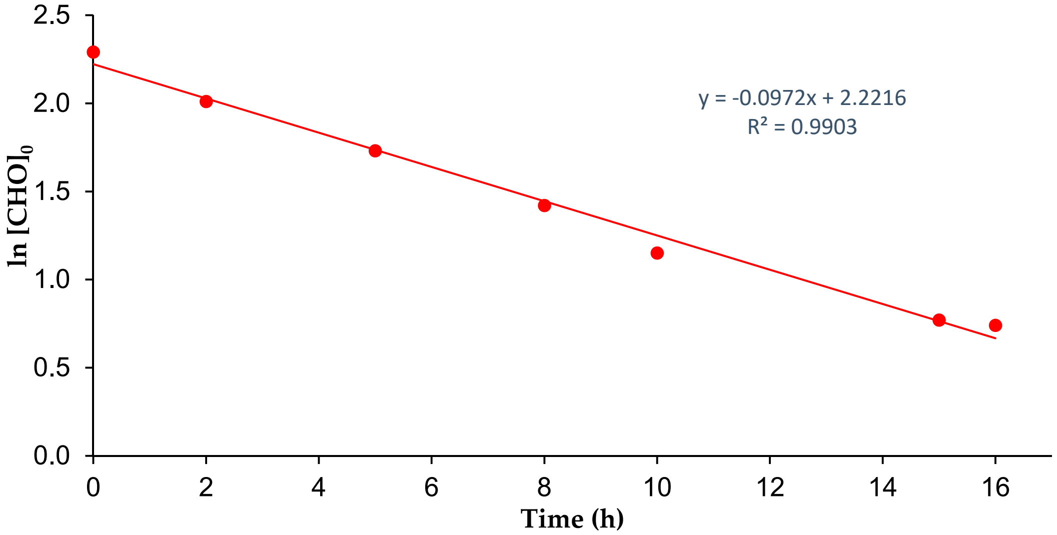

**Figure S6.** Pseudo first-order kinetic plot for the ring-opening copolymerisation of cyclohexene oxide and carbon dioxide catalysed by complex **4** at 70 °C and 10 bar $CO_2$.

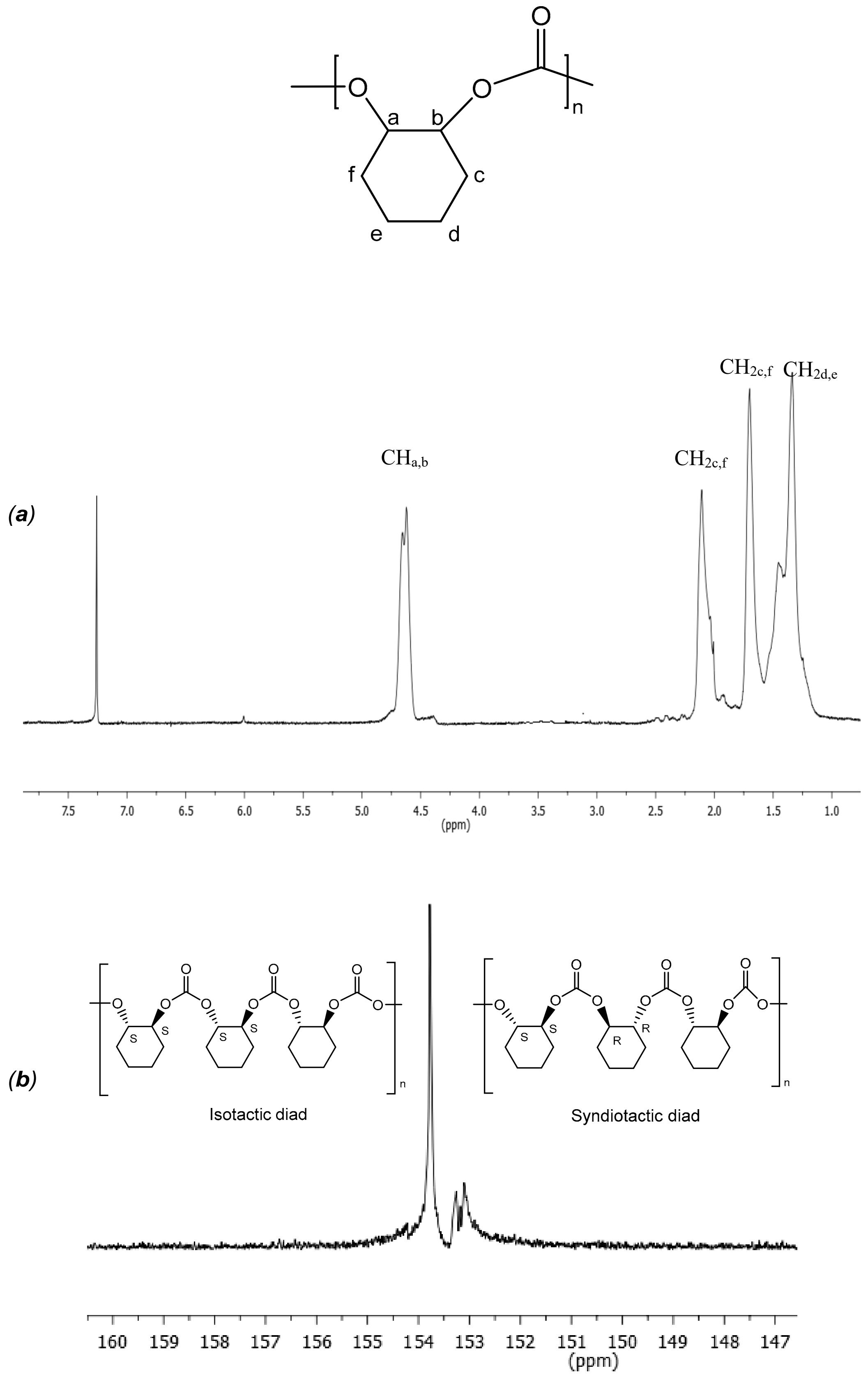


**Figure S7**: (*a*) $^{1}H$ NMR and (*b*) $^{13}C$-{$^{1}H$} NMR spectrum of poly(cyclohexene carbonate) sample (Table 4, entry 5) using complex **4** as catalyst at 70 °C and 10 bar $CO_2$.
